# The complete chloroplast genome sequence of *Spiraea*×*vanhouttei* (Briot) Zabel (Rosaceae)

**DOI:** 10.1080/23802359.2022.2052369

**Published:** 2022-03-17

**Authors:** Min-min Chen, Rui-hong Wang, Hong-kun Sha, Ming-zhi Liu, Jian-quan Tong, Qiu-Ling He

**Affiliations:** a College of Life Sciences and Medicine, Zhejiang Province Key Laboratory of Plant Secondary Metabolism and Regulation, Zhejiang Sci-Tech University, Hangzhou, China; b Zhejiang Chun'an County Agricultural and Rural Development Service Center, Hangzhou, China

**Keywords:** *Spiraea*×*vanhouttei*, *Spiraea*, chloroplast genome, phylogeny

## Abstract

*Spiraea*×*vanhouttei* (Rosaceae) is a frequently planted *Spiraea* species that is distributed in Shandong Province, Jiangsu Province, and Guangdong Province, China. The first complete chloroplast genome of *Spiraea*×*vanhouttei* was determined and described in this study. The genome is 155,957 bp in length and contained 129 encoded genes in total, including 84 protein-coding genes, eight ribosomal RNA genes, and 37 transfer RNA genes. The phylogenomic analysis showed that *Spiraea*×*vanhouttei* was closely related to *Spiraea blumei* according to the current sampling extent.

The genus *Spiraea* L. (Rosaceae) encompasses more than 100 species. *Spiraea*×*vanhouttei* (Briot) Zabel (1884) is one of *Spiraea* L., commonly named as Van Houtte spiraea or Vanhoutte spirea (KůdEla 2008). *Spiraea*×*vanhouttei* is a hybrid between *Spiraea cantoniensis* Lour. and *Spiraea trilobata* L., mainly distributed in Shandong Province, Jiangsu Province, and Guangdong Province, China (Harris 1917). Additionally, due to its conspicuous, luxuriant, and showy flowers, *Spiraea*×*vanhouttei* are frequently used as ornamentals and cut flowers (Yücesan et al. 2018). It has been used to treat diarrhea, as an astringent, anti-bacterial, and early source of salicylic acid, which is aspirin's main component. In this study, the first complete chloroplast genome of *Spiraea*×*vanhouttei* was determined and described. The study will provide potential genetic information for systematics, molecular ecology, and phylogenetic analyses in the genus *Spiraea*.

The leaves of *Spiraea*×*vanhouttei* were collected from Lin’an, Zhejiang, China (GPS: E119°42′44.85″, N30°16′27.04″). The specimen and extracted DNA were deposited at public herbarium of College of Life Sciences and Medicine, Zhejiang Sci-Tech University (Zhejiang Province Key Laboratory of Plant Secondary Metabolism and Regulation, http://sky.zstu.edu.cn/, Identifier: Qiu-Ling He, qlhe@zstu.edu.cn) under the voucher number ZSTULSM0002. The total genomic DNA was extracted from its silica dried leaves using DNA Plantzol Reagent (Invitrogen, Carlsbad, CA) following the manufacturer’s instructions. The plastome sequences were generated using the Illumina HiSeq 2500 platform (Illumina Inc., San Diego, CA). In total, ca. 23.9 million high-quality clean reads (150 bp PE read length) were generated with adaptors trimmed. These clean data were *de novo* assembled to complete chloroplast genome using GetOrganelle (Jin et al. 2020), Geneious v11.1.5 (Biomatters Ltd, Auckland, New Zealand) (Drummond 2012) was used to annotate the genome with *Spiraea insularis* plastome (GenBank: MT412405) as a reference.

The full length of *Spiraea*×*vanhouttei* chloroplast sequence (GenBank accession no. MZ981785) is 155,957 bp, consisting of a large single-copy region (LSC with 84,384 bp), a small single-copy region (SSC with 18,893 bp), and two inverted repeat regions (IR with 26,340 bp). The overall GC content of *Spiraea*×*vanhouttei* chloroplast genome was 36.8% and the GC content of the LSC, SSC, and IR regions was 34.6%, 30.4%, and 42.5%. The genome contains a total of 129 genes (84 protein-coding genes, eight rRNA genes, and 37 tRNA genes). Seventeen genes had two copies, which were comprised of six PCG genes (protein-coding genes) (*ndhB, rps7, ycf2, rpl2, rpl23, rps12*), seven tRNA genes (*trnI-CAU, trnV-GAC, trnI-GAU, trnA-UGC, trnR-ACG, trnN-GUU, trnL-CAA*), and all four rRNA species (*rrn16, rrn23, rrn4.5, rrn5*). In the genome, 11 protein-coding genes (*atpF, rpl2, ndhB, rps16, rpoC1, clpP, rpl16, petD, petB, rps19, ndhA*) had one intron, *rps12* and *ycf3* gene contained two introns.

To confirm the phylogenetic position of *Spiraea*×*vanhouttei*, we obtained 10 published complete full-length chloroplast genomes of Rosaceae from NCBI. *Elaeagnus multiflora* was used as an outgroup for constructing the phylogenetic tree. The entire full-length chloroplast genome sequence alignment was conducted using MAFFT v7.3 (Katoh and Standley 2013). The phylogenetic relationship was analyzed on the complete chloroplast genomes with maximum-likelihood (ML) method using IQTREE v1.6.7 (Nguyen et al. 2015). The ML phylogeny was inferred under the best-selected TVM + F+R3 model and 5000 bootstrap replicates. The phylogenetic tree revealed that *Spiraea*×*vanhouttei* was closely related to *Spiraea blumei* with high support according to the current sampling extent (Figure 1).

**Figure 1.**
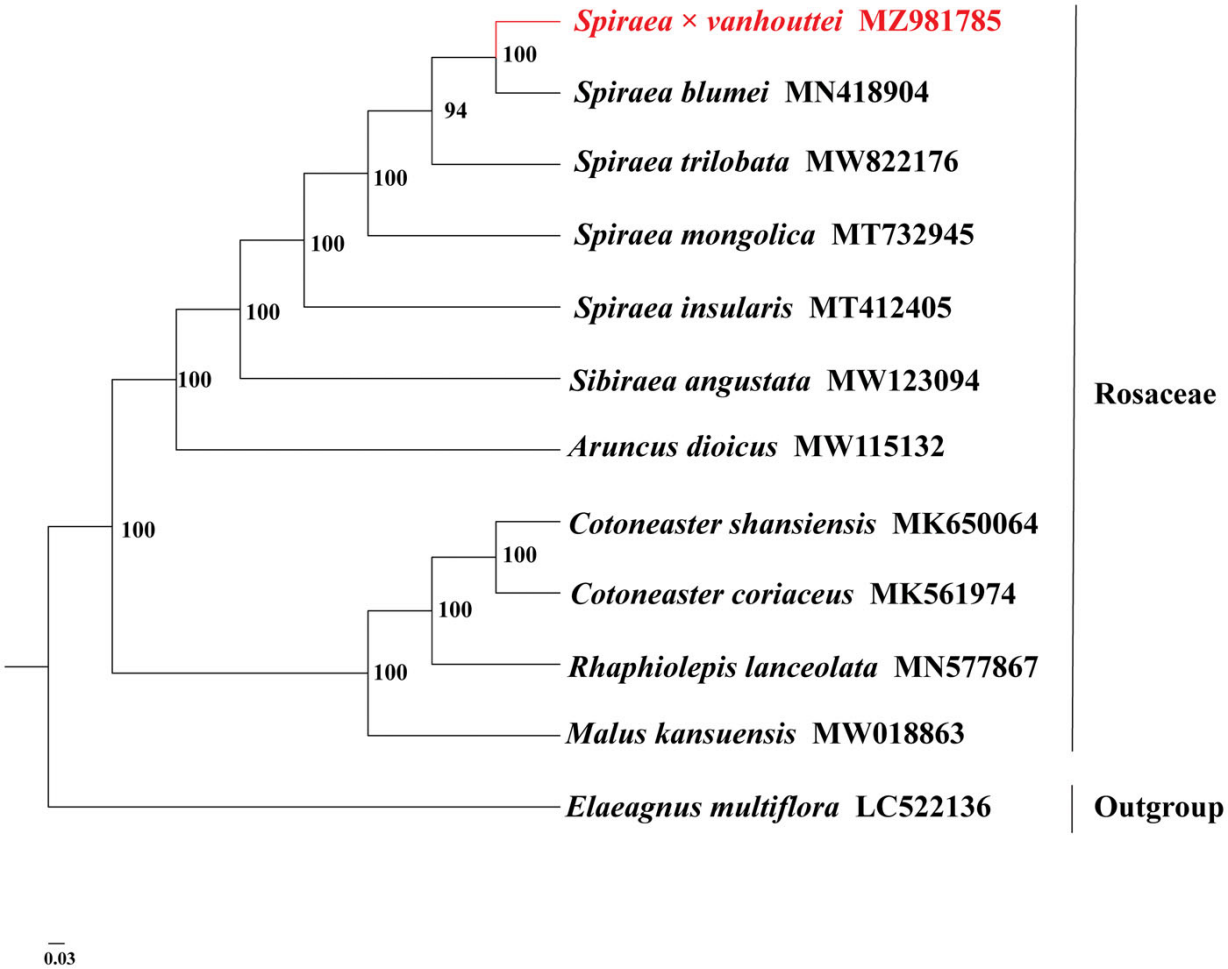
The phylogenetic tree based on 11 complete chloroplast genome sequences in Rosaceae and one complete chloroplast genome sequences in Elaeagnaceae using maximum-likelihood (ML) method (accession numbers were listed behind each taxon. Statistical support values are shown on nodes).

## Data Availability

The genome sequence data that support the findings of this study are openly available in GenBank of NCBI (https://www.ncbi.nlm.nih.gov) under the accession no. MZ981785. The associated BioProject, SRA, and Bio-Sample numbers are PRJNA759557, SRR15686219, and SAMN21169300, respectively.
